# The Effect of the COVID-19 Pandemic on Saudi Adults' Behavior Regarding Food Literacy and Food Consumption

**DOI:** 10.7759/cureus.27878

**Published:** 2022-08-11

**Authors:** Majid M Alkhalaf, Khlood A Bookari, Jamila Arrish, Ghadir A Fallata, Omar A Alhumaidan, Shihana A Alakeel, Norah A AlBuayjan, Sarah M Alkhunein, Budur M Bin Obaydan, Aeshah A Alshaya

**Affiliations:** 1 Nutrition, Saudi Food and Drug Authority, Riyadh, SAU; 2 Nutrition, Faculty of Applied Medical Sciences, Taibah University, Riyadh, SAU; 3 Nutrition, University of Wollongong, Wollongong, AUS; 4 National Nutrition Committee, Saudi Food and Drug Authority, Riyadh, SAU

**Keywords:** behavior food literacy, food security, covid-19, pandemic, food consumption, food literacy

## Abstract

Background

The coronavirus pandemic has forced worldwide closures, especially of restaurants closed, which partly contributed to people all over the world changing the way they choose and prepare foods.

Objective

The objective of this study was to compare changes in behavioral food literacy (planning, selecting, and preparing food) and food consumption before and during the COVID-19 crisis in the Kingdom of Saudi Arabia (KSA).

Design

This was a cross-sectional study, with data from an online survey made in UAntwerpen Qualtrics Accounts and collected from April-June 2020. The study was part of the International Corona Cooking Survey.

Results

There were 2788 respondents (83%, n = 2323 females) who reported that the COVID-19 home lockdown had positively affected all their behavioral food literacy components (all p<0.05) except feeling confident about cooking a variety of healthy meals in which the difference was not significant (p>0.05); however, its impact on their food consumption was varied. There was a noticeable positive increase in fruit intake (Z= -3.330, p=0.001) and a noticeable positive decrease in processed meat (Z= -11.375, p<0.001) and sweetened drinks consumption (Z= -2.403, p<0.05). There were simultaneously noticeable adverse effects represented in the reduction in the consumption of the vegetable group (Z= -3.447, p=0.001) and an increase in sweets consumption (Z= -2.268, p<0.05). However, the overall impacts of these changes as measured by the Hedges’ g measure indicated a small effect (Hedges’ g = 0.04, 95% CI (-0.07, -0.16)).

Discussion and conclusions

Even though the pandemic may have created a sense of responsibility for one's health and increased people's nutritional awareness, the Saudi population may be still a long way from having healthy eating habits. Public health campaigns need to increase the population's level of nutritional awareness, educate them about the meaning of healthy eating, and how they can achieve that by advocating the national dietary guidelines and providing reliable and accurate information by authorized official bodies.

## Introduction

In the world of infectious diseases, the worst possible scenario that may occur is a pandemic. When an epidemic passes the nation's borders and continues to spread globally, that is when the epidemic formally becomes a pandemic [1]. The world has previously encountered many infectious diseases. Spanish flu in 1918, human immunodeficiency virus (HIV) in the early 1980s, Hantavirus in 1993 in the US, SARS in 2002 in the Guangdong province of China, and MERS in 2012 in Saudi Arabia are some examples [1]. More recently, in late 2019, the novel severe acute respiratory syndrome coronavirus 2 (SARS‑CoV‑2), generally known as COVID-19, broke out in the city of Wuhan in the Hubei province in the center of China [2]. Shortly afterward, on 11 March 2020, following its fast and significant escalation in 114 countries, the World Health Organization (WHO) declared COVID-19 a global pandemic leading to a new acute severe respiratory syndrome disease [3]. In response, most national governments worldwide have taken highly restrictive measures to limit and avoid the spread of this new virus [4]. These measures include but are not limited to the closure of social meeting areas, such as schools and places of worship and places of work, restraints on public transportation, bans on major public gathering events, restrictions on the movement of individuals, and the postponing of local and international flights [4].

Similarly, the Saudi government has taken preventive measures after the first case of COVID-19 was identified in March 2020 and imposed guidelines on its citizens [5]. This trend echoes the global trend of virus outbreaks. As of 25 July 2022, 808,053 confirmed cases with over 9,240 deaths due to the COVID-19 virus reported in Saudi Arabia [6]. Indeed, a greater number of measures have been focused on entire city and region lockdown as the only solution in many instances to counter the pandemic; such as to not leave home except for meeting their basic needs (for example getting out because of medical emergencies), doing some work (especially for those who work in the health and national security sectors), and purchasing essential items like food [5]. Although these measures were considered crucial to halt the spread of COVID-19, they imposed considerable disruption to health, economy, agri-food systems, people's social lives, behavioral food literacy, and food consumption [7]. The term "behavioral food literacy" was used to describe the set of skills, knowledge, and behaviors of daily processes related to healthy eating and is defined by its components [8]. In this study, behavioral food literacy is defined as the ability to plan, select, and prepare food. The challenge for public health, food systems, and businesses is remarkable.

After the imposition of these preventive measures, people spent more time than usual in their homes, and hence they had more time for cooking and snacking. Restrictions on freedom of movement may have also affected the practice of food provision and the access and availability of specific food items [9]. Notably, good nutrition and a well-balanced diet are essential for sound health and well-being, particularly in illness periods when the immune system is challenged [10]. Furthermore, the boredom, anxiety, and stress provoked by the lockdown preventive measures are likely to increase emotional eating, and so forth. Expected changes in people's behavioral food literacy and consumption due to the COVID-19 pandemic are attributed to the lockdown and social isolation directives and confusion about what is going to happen in the near future. Understanding food planning, selecting, preparing, and consumption behaviors is beneficial not only for understanding how people's actions shift and adapt during crisis times but also for providing practical support in emergency response efforts.

In this context, some studies have been established to estimate the impact of COVID-19-related lockdown measures on the food consumption habits of adults at international and national levels [11]. Most of these studies reported a change to a certain extent in people's dietary habits after the COVID-19 pandemic [12]. The reported dietary changes varied from one country to another, with the lowest percentage (17%) reported in the Netherlands [13] and the highest (70%) in Canada. In the meantime, the COVID-19 crisis has yielded some benefits. While the majority (83%) of Dutch people did not eat either "(un)healthier" or larger quantities of food, a shift toward a healthier diet compared to people's usual habits was reported among adults living in Poland and Qatar during the lockdown [1,13]. Unfortunately, most other studies have noted otherwise, indicating that many people started eating more, snacking more, exercising less, and gaining weight due to the lockdowns [12]. It remains to be seen if these effects apply to other nations, given the local variation in dietary habits and the magnitude of the lock-down measures. Although earlier studies examined the pandemic's effects on Saudi adults' nutritional behaviors, the impact on the planning, selecting, and preparing of healthier foods has not yet been explored in Saudi Arabia [11,14].

The International Corona Cooking Survey project organized by the University of Antwerp in Belgium was established to explore the impact of COVID-19-related lockdown measures on eating habits, shopping and cooking behaviors, and media habits [15]. It is an international, cross-sectional online survey conducted in 38 countries worldwide. Cross-sectional data from this survey have recently been presented concerning the impact on the planning, selecting, and preparing of healthier foods as elements of the behavioral food literacy concept [16].

This study is part of a larger collaborative international study to investigate changes in the behavioral food literacy and dietary behavior among the adult population [16]. This paper aims to provide a description of the effect of the lockdown measures on Saudi adults' dietary patterns as well as on their behaviors concerning planning, selecting, and preparing healthier foods [16].

## Materials and methods

Study design and participants

An international cross-sectional online survey was conducted to assess the impact of COVID-19 on cooking behavior, and food consumption in 38 countries. The National Nutrition Committee (NNC) in the Saudi Food and Drug Authority (SFDA), in collaboration with the University of Antwerp, modified and translated this study questionnaire into Arabic to make it relevant and culturally appropriate for the Saudi population. An independent translator to ensure the accuracy of the translation back-translated the questionnaire. The components of the questionnaire included a selection of demographic and dietary behavior questions for the period before and during the lockdown (planning, selecting, and preparing food). Likewise, in collaboration with other agencies and academic experts, the Arabic version of the questionnaire was approved and used by all Arabic-speaking countries in the international study. The respondents were recruited through convenience sampling. Since the target population of this paper is adults in Saudi Arabia during the period of COVID-19 restrictions, participants were eligible if they were >18 years and residing in Saudi Arabia during that period. Saudi adult respondents residing outside Saudi Arabia were excluded from the analysis of this paper.

Confidentiality of information and research ethics

Study information collected from participants was kept strictly confidential by password-protected files on secure servers (in a Qualtrics survey account), and no information was disclosed to any party without explicit written consent from the principal investigator of the study. Ethical approval for this study was obtained from the ethics committee for the Social Sciences and Humanities at the University of Antwerp in Belgium, as well as the research ethics committee at Taibah University in the Kingdom of Saudi Arabia (project code SREC/AMS 2020/62/CND and date of approval on May 2, 2020).

Measurement tools:

Food literacy behavior is usually measured by a validated scale that contains 11 items capturing three main domains of “planning”, “selecting”, and “preparing” healthier foods, which was used in this study. The scale items measured the behavior by using Likert scales (1 = never; 2 = very rarely; 3 = rarely; 4 = sometimes; 5 = frequently; 6 = very frequently; 7 = all the time). Food groups' consumption frequency was measured by using Likert scales (1 = never; 2 = Less than one serving per week; 3 = one serving per week; 4 = two to four servings per week; 5 = five to six servings per week; 6= one serving per day; 7 = two or more servings per day). The participants were asked to answer the questions related to behavioral food literacy and food consumption twice; reporting their behavior before and during the COVID-19 pandemic.

Statistical analysis

The data of this study were analyzed using the Statistical Package for Social Sciences (SPSS- version 16.0). The Shapiro-Wilk test was used to test the normality of the data. Whereas, the Wilcoxon signed-rank test was used to compare the means of behavioral food literacy and food consumption items before and during the COVID-19 pandemic. The data presented as means and standard deviation. The effect size of the changes in behavioral food literacy and food consumption by using the Hedges' g measure. The measure interprets the effect size using the value of (0.2) represents a weak effect, (0.5) a moderate effect, and (0.8) a strong effect. The significance level was set at a p-value of < 0.05.

## Results

Participants

Table 1 shows the characteristics of the participants. The total number of participants with complete responses was 2788, of which the majority were female (83%, n = 2323). More than half of the participants (3156.5 %, n = 1576) were aged between 19 and 29 years old. This was reflected in the education level of the participants where more than 60% of the participants were holders of a bachelor's degree or equivalent. In terms of the employment status of the participants, there was a decrease in the percentage of the participants who were employed during the pandemic compared to those who were employed before the pandemic by 10% (36%, n = 1002 versus 26%, n =736). It was also noticed that the rate of unemployment among the participants increased by 13% (29%, n = 810 versus 42%, n = 1174) during the pandemic compared to the period before it.

**Table 1:** Characteristics of study participants (n=2788)

Characteristics	Total (n=2788)	
	n	(%)
Age (years) 19-29	1576	(56.5)
30-39	730	(26.2)
40-49	326	(11.7)
50 and more	156	(5.6)
Total	2788	
Gender	n	(%)
Female	2323	(83.3)
Male	465	(16.7)
Total	2788	
Education	n	(%)
Under a high school diploma	92	(3)
High school diploma or equivalent	678	(24)
Bachelor’s degree	1767	(63)
Master’s degree	192	(6.8)
Doctorate degree	59	(2)
Total	2788	
Employment status Before COVID-19	n	(%)
Student	976	(35)
Employed	1002	(36)
Unemployed	810	(29)
Total	2788	
Employment status during COVID-19	n	(%)
Student	878	(31)
Employed	736	(26)
Unemployed	1174	(42)
Total	2788	

The impact of COVID-19 on behavior food literacy components

The impact of COVID-19 on the food literacy components (i.e., planning, selecting, and preparing) as reported by the participants in the study is presented in Table 2 using Wilcoxon Signed Ranks test. As shown in the table, there was a significant difference in all food literacy behavior components related to planning, selecting, and preparing food during COVID-19 compared to the period before the pandemic (all p<0.05) except in regard to feeling confident about cooking a variety of healthy meals in which the difference was not significant (p>0.05). Overall, there was an improvement in all the food literacy components related to planning, selecting, and preparing food during COVID-19 in comparison to the period before the pandemic. Participants did not only plan their meals ahead of time and make a shopping list before doing their shopping (p<0.001), but the results also showed a remarkable improvement in the process of planning healthier meals by the participants via attempting to incorporate all the food groups (Z= -9.699, p <0.001). The study participants thought more about healthy food when deciding what to eat (Z= -5.287, p<0.001), and their use of nutritional panels and other parts of food labels in their selection of food also increased during the COVID-19 pandemic (Z= -5.272- and 7.786 respectively, p<0.001). Participants also reported being more confident in using money to purchase healthy food during the crisis. Furthermore, they reported an increase in the rate of: cooking more healthy meals at home by using healthy ingredients (Z=-2.221, p<0.05), trying new recipes (Z= -3.334, p=0.001), and changing them to be healthier (Z=-5.987, p<0.001).

**Table 2:** Behavioral food literacy components before and during COVID-19 # Data presented as means and standard deviation (SD). *Value was significantly different between groups (P <0.05) based on the Wilcoxon signed-rank test

P-value			During COVID-19		Before COVID-19		Behavioral Food literacy components
			SD	Means	SD	Means	
0.000			(1.86)	3.99	(1.67)	3.70	Plan meals ahead of time
0.000			(1.95)	4.80	(1.94)	4.64	Make a list before you go shopping
0.000			(1.80)	4.00	(1.78)	3.73	Plan meals to include all food groups (A varied diet)
0.000			(1.77)	4.13	(1.74)	3.98	Think about healthy choices when deciding what to eat
0.000			(1.85)	4.17	(1.81)	3.93	Feel confident about managing money to buy healthy food
0.000			(1.89)	3.49	(1.84)	3.36	Use the nutritional information panel to make food choices
0.000			(1.84)	3.54	(1.78)	3.34	Use other parts of the food label to make food choices
0.026			(1.78)	4.41	(1.74)	4.35	Cook meals at home using healthy ingredients
0.893			(1.84)	4.60	(1.86)	4.58	Feel confident about cooking a variety of healthy meals
0.001			(1.70)	4.63	(1.66)	4.54	Try a new recipe
0.000			(1.72)	4.19	(1.68)	4.05	Change recipes to make them healthier

**Figure 1:**
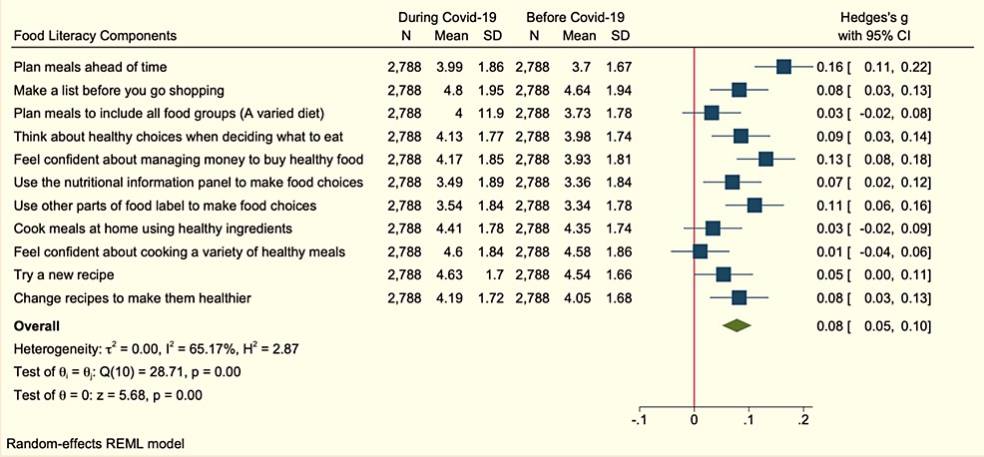
Components of behavioral food literacy Shows the plot and values of Hedges’ g effect size and 95% confidence interval (CI) for changes in behavioral food literacy before and during COVID-19 in the kingdom of Saudi Arabia. REML: restricted maximum likelihood

Although there was a noticeable improvement in most of the behavioral food literacy components related to planning, selecting, and preparing food during COVID-19 among the study participants, the overall effect of this improvement measured by the Hedges’ g measure was less than 0.2 indicating a small effect (Hedges’ g = 0.08, 95% CI (0.05-0.10)) as shown in Figure 1.

The impact of COVID-19 on food consumption

Table 3 shows the impact of COVID-19 on food consumption in Saudi Arabia. Using the Wilcoxon signed-ranks test, there was a significant difference in the consumption of fruit, vegetables, processed meats, unprocessed meat, unsweetened (such as water, coffee, and tea), and sweetened beverages during COVID-19 in comparison to the period that preceded the pandemic. The results indicate a slight positive increase in the consumption of fruit (Z=-3.330, p=0.001) but a decrease in the consumption of vegetables simultaneously (Z= -3.447, p=0.001). There was also an increase in the consumption of unprocessed red meat (Z=-6.279, p<0.001) while the consumption of processed meats (i.e. red meat/fish/poultry/vegetarian alternatives) decreased (Z= -11.375, p<0.001). Although, the consumption of sweet snacks increased (Z= -2.268, p<0.05) slightly during COVID-19, a remarkable increase in the consumption of unsweetened beverages (Z= -8.214, p<0.001) was noted. This was accompanied by a minor decrease in the consumption of sweetened beverages (Z= -2.403, p<0.05). The consumption of unprocessed fish and poultry, legumes, nuts or nut spread, whole and refined grains milk and dairy products, plant-based drinks, and salty snacks undergo no changes (all p>0.05).

**Table 3:** The participants' food group consumption before and during COVID-19 # Data presented as means and standard deviation (SD). *Value was significantly different between groups (P <0.05) based on the Wilcoxon signed-rank test.

P-value	During Covid-19		Before Covid-19		Food Groups
	SD	Means	SD	Means	
0.001	(1.57)	3.78	(1.52)	3.70	Fruit
0.001	(1.54)	4.32	(1.51)	4.40	Vegetables
0.867	(1.40)	3.32	(1.31)	3.33	Legumes
0.423	(1.59)	3.20	(1.50)	3.23	Nuts or nut spread
0.000	(1.61)	3.10	(1.67)	3.46	Processed meats (red meat/fish/poultry/vegetarian alternatives)
0.417	(1.51)	2.57	(1.39)	2.55	Unprocessed fish
0.785	(1.62)	3.89	(1.62)	3.90	Unprocessed poultry
0.000	(1.50)	3.50	(1.44)	3.36	Unprocessed red meat
0.772	(1.54)	2.06	(1.48)	2.05	Unprocessed vegetarian meat alternatives
0.023	(1.56)	4.10	(1.46)	4.06	Sweet snacks
0.315	(1.63)	3.42	(1.53)	3.45	Salty snacks
0.253	(1.65)	3.79	(1.58)	3.82	Whole grains
0.608	(1.54)	4.12	(1.48)	4.12	Refined grains
0.792	(1.74)	4.19	(1.66)	4.20	Milk
0.573	(1.51)	4.64	(1.47)	4.66	Dairy products
0.108	(1.77)	2.49	(1.74)	2.52	Plant-based drinks
0.000	(1.78)	5.13	(1.71)	2.35	Unsweetened beverages
0.016	(2.00)	3.85	(1.95)	3.92	Sweetened beverages

**Figure 2:**
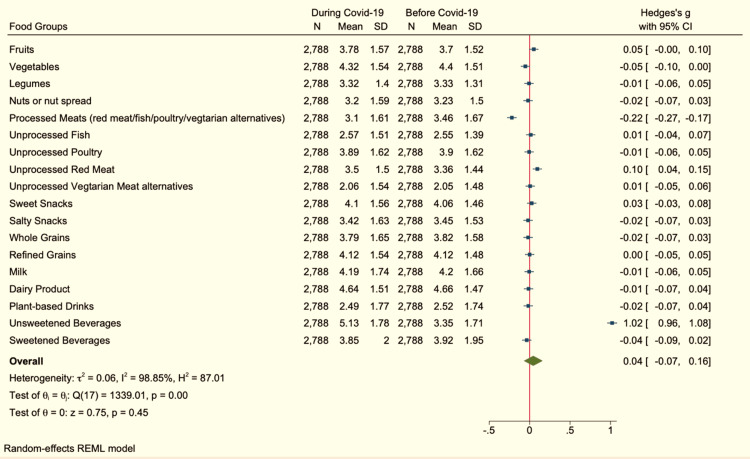
Food consumption Plot and values of Hedges’ g effect size and 95% confidence interval (CI) for changes in food consumption before and during COVID-19 in the kingdom of Saudi Arabia. REML: restricted maximum likelihood

Despite the significant changes found in the consumption of some foods, the effect of these changes measured by the Hedges’ g measure was less than 0.2 pointing to a small effect of COVID-19 on food consumption among the participants (Hedges’ g = 0.04, 95% CI (-0.07, -0.16)) as shown in Figure 2.

## Discussion

This observational population-based study aimed to explore how the COVID-19 pandemic has impacted behavioral food literacy and food consumption among a sample of Saudi adult residents. Our study is one of Saudi’s first studies to provide a snapshot of the immediate impact that COVID-19 has had on planning, selecting, and preparing healthier foods among Saudi participants after seven weeks of home lockdown. There were 2788 respondents (83%, n = 2323 females) and it was found that the COVID-19 home lockdown positively affected all behavioral food literacy components; however, its impact on food consumption was varied. The increase in fruit group intake and the decrease in processed meat and sweetened drinks consumptions were among the most noticeable positive results. The reduction in consumption of the vegetable group and the increase in sweets consumption were among the noticeable adverse effects. Additionally, the effect size of these changes exhibited. The overall impacts measured by the Hedges’ g measure indicated a small effect.

The Saudi population has reported an improvement in most behavioral food literacy components after the lockdown measures implemented in response to the COVID-19 pandemic. There have been noticeable changes in how participants plan their meals in advance and create a shopping list before going shopping. This finding is consistent with the results of the Corona Cooking Survey’s study, which was conducted in 38 countries worldwide and the research conducted in Qatar [14,16]. Personal perceptions about having more time at home due to stay-at-home policies during the COVID-19 crisis may play a significant role in explaining this outcome since time was one of the most frequently reported hindrance factors for planning, choosing, and preparing healthy foods [17]. This result is regarded to be one of the beneficial effects of the preventive lockdown measures, taking into account the negative consequences associated with time constraints in terms of adopting healthy eating behaviors. Long work hours (more than 40 hours per week) were associated with many health-related time beliefs and behaviors. Being too busy to eat healthfully, finding it difficult to allocate time to sit down and eat a meal, and the belief that thinking of healthy eating takes too much time are some reported examples [18]. In addition, extended work hours have been associated with a lack of concern for dietary balance, irregular lunches, and late dinners [19]. The extra time people spent at home during the pandemic may have had a beneficial effect on their dietary behaviors. It provided an opportunity for them to experience the benefits of changing their eating habits. If people felt a favorable effect, they may continue to do so in the future after the pandemic ended. Hence, interventions that aim at the community level and prompt people to eat better should consider this result and provide simple tips on planning and preparing healthy meals while working full time when the pandemic ends.

Another beneficial effect the COVID-19 pandemic may have had on people’s dietary habits is that people might have become more aware of the importance of maintaining good health by adopting healthy eating behaviors. According to our study’s participants, there was a desire to improve their diet in response to the COVID-19 pandemic. The participants reported notable improvements in meal planning by incorporating all food groups and focusing on nutritious meals when determining what to eat. They paid more attention to nutritional panels and other food labeling methods when selecting foods during the COVID-19 pandemic. Moreover, participants indicated that during the pandemic, they reported an increased desire to cook at home and tried to create more healthy meals by utilizing healthy ingredients, trying new recipes, and creating a healthy food version. Unlike other studies, this finding is comparable with findings from studies conducted in the Netherlands, Poland, Spain, and Qatar, where their participants reported a change toward a better diet during the lockdown compared to their regular behaviors [12].

The rapid spread of the virus, the scarcity of reliable information about it, and the high number of deaths caused by the infection, particularly in the early stages of the pandemic, made people aware of the gravity of the situation and made people realize how fragile life is and how important health is. The unusual circumstances that the world has faced since the pandemic has motivated people to prioritize their health to limit their risk of infection by adopting healthy eating habits [20]. This behavior is explicable by the idea of health consciousness, which refers to an individual’s level of concern for their health [21]. More health-conscious individuals are more likely to have good behaviors, which serves as the foundation for individuals to take healthy actions. As a result, one may claim that the pandemic provoked people’s health consciousness [22]. However, it is important to note that while this aspect may be viewed as positive and important in bringing about change, it could be insufficient, particularly when it comes to modifying eating habits. According to the Transtheoretical Model's stages theory, people may be in the contemplation stage, and the corona pandemic may have resulted in the formation of intentions for people to take action and a plan to correct their diet [23]. As a result, people may require guiding and educational plans to enable them to do so in the proper manner, so they can positively progress into the next stages.

The COVID-19 epidemic has caused a devastating blow to the global economy, and despite its strength, the Saudi economy was not immune. However, the pandemic has created a wise spending culture in Saudi society. The strategic plans developed by the Saudi government have been focused on meeting the fundamental life necessities for members of society throughout the pandemic while also considering the long-term sustainability of the national economy. Food is one of the most crucial necessities of life that the government has worked hard to ensure is available to all citizens. These efforts may explain the results of our study, as the participants reported being more confident in using money to purchase healthy food during the crisis. The participants’ financial confidence might be one of the most visible results of the Saudi government’s efforts to mitigate the negative impact of COVID-19 on food abundance. The ministries of commerce and finance have collaborated to manage and monitor food costs and provide financial assistance to low-income families and small businesses affected by the pandemic [24]. For people in our study, another explanation for their high financial confidence in purchasing foods could be that preventive lockdown measures may have affected the public’s food purchasing habits. Many people have been forced to shop online, either out of fear of interacting with other people in public spaces and thus exposing themselves to the risk of virus infection, or because the hours allowed for shopping are inconvenient for them, making online shopping the more appropriate and convenient option. Online shopping may offer an advantage over in-store shopping in that it enables consumers to purchase only what they require, or at the very least what they have preselected during meal planning, and lessens unhealthy impulse purchases [25,26]. The converse of what occurs when a person shops at a supermarket, when he purchases more than he requires or intends, is influenced by supermarkets' marketing strategy [27]. Online shopping may last far beyond the COVID-19 pandemic. As a result, public health campaigns should focus on increasing people’s awareness about wise usage of online shopping to acquire control over the quality and quantity of food they buy.

In contrast to the remainder of the behavioral food literacy components examined in this study, participants’ confidence in their ability to prepare a range of healthy meals showed no significant change due to the pandemic. This finding could be explained by the fact that most of our study participants were women who may have had this sense of confidence before the pandemic. This is because women are known to be not only familiar with home cooking but also responsible for it even before the pandemic began, especially in Arabic countries [28]. Experience and familiarity create a sense of self-efficacy (confidence) in performing tasks such as cooking foods [29]. Therefore, their practice of cooking during the pandemic did not significantly change their confidence. Cooking at home is an excellent approach to gaining control of a person’s nutrition as well as his/her wallet [28]. However, perceived confidence in food preparation due to expertise and familiarity does not necessarily translate into healthier eating behaviors. In addition, strong intentions to cook healthy meals at home are not always followed through. In our study, the pattern of dietary consumption has reflected the previously stated concepts. There were two opposite dietary change patterns derived from the analysis. The healthy changes included increases in the consumption of fruit, unprocessed meat, and unsweetened beverages (such as water, coffee, and tea), whereas consumption of processed meats (i.e. red meat/fish/poultry/vegetarian alternatives) decreased. The unwanted effects included a drop in vegetable intake and an increase in the consumption of sugary snacks. This inconsistent dietary pattern was not different from what was reported in previous studies conducted in Saudi Arabia [30]. 

## Conclusions

Although the pandemic may have created a sense of responsibility for one's health and increased people's nutritional awareness, the studied Saudi adult population might be still a long way from having healthy eating habits, as evidenced by the inconsistent changes in their food intakes that do not reflect healthy eating. Accordingly, we can say that public health campaigns need to focus on increasing people’s level of nutritional awareness and educating them about the meaning of healthy eating and how it can be achieved. People need to know the recommendations of national dietary guidelines and what is the specific amount they need to consume from each food group. Additionally, because there is a flood of false and misleading information spread via social media and traditional media channels during the pandemic, authorized official bodies must speak out and provide people with reliable and accurate references that enable them to obtain accurate and reliable information.

While it is reasonable to believe that the pandemic has contributed to people desiring healthier eating habits, other factors such as the inconsistent implementation of dietary changes or the differences in how participants expressed the meaning of the term "healthy eating" should be taken into account. One of these potential factors is a lack of nutritional information and an inadequate understanding of what constitutes a healthy diet, which we did not survey as this falls outside the scope of our study. 

People’s precise understanding of dietary recommendations can help them make acceptable food choices and attain a healthy diet. It also helps them reject misleading or inaccurate advice from family, friends, and social media. However, for some people, stay-at-home regulations can exacerbate anxiety symptoms.

Strength and limitations

There are several strengths of this study. First, it is the first study in examining the impact of planning, selecting, and preparing healthier foods before and during the COVID-19 imprisonment in Saudi Arabia under a new situation such as the lockdown caused by the COVID-19 pandemic. Second, the online survey was translated into Arabic and reviewed by a group of researchers in the NNC at SFDA which was later used in several Arab countries. This made the survey more accessible to the Arabic and Saudi population and reduced any chance of ambiguity in the questions of the survey. Moreover, the online survey provided a rapid and cost-efficient way of obtaining self-reported information about dietary behaviors during the COVID-19 pandemic in Saudi Arabia. It was a useful way to collect a large number of responses when it had been relatively impossible to have face-to-face interviews due to the lockdown.

However, some limitations should also be acknowledged. For example, the use of an online survey may not be representative of the entire population of quarantined people in Saudi Arabia. Another limitation is that respondents were predominantly women (around 83.3% of the participants). In addition, the selection bias cannot be ruled out, indicated by the low participation of people who were more than 50 years (5.6%) which could be due to the reason that this generation is less likely to use smart technologies and is unable to access the internet. This could have been an obstacle for this group to participate in the study. Furthermore, self-reported information about food consumption behavior carries the risk of bias as participants are more likely to report positive than negative behaviors.
